# Contribution of environmental and biological factors to bacterial community structure and stability in a subalpine lake

**DOI:** 10.1007/s42995-024-00256-8

**Published:** 2024-10-30

**Authors:** Ping Guo, Cui Li, Jinxian Liu, Tiehang Wu, Baofeng Chai

**Affiliations:** 1 Central Laboratory, Changzhi Medical College, Changzhi, 046000 China; 2 Shanxi Key Laboratory of Ecological Restoration On Loess Plateau, Institute of Loess Plateau, Shanxi University, Taiyuan, 030006 China; 3 Faculty of Environment Economics, Shanxi University of Finance and Economics, Taiyuan, 030006 China; 4 Department of Biology, Georgia Southern University, Statesboro, GA 30460-8042 USA

**Keywords:** Bacterial community, Environmental factors, Biological factors, Community structure, Network stability

## Abstract

Bacterial community play an essential role in regulating water quality and the global biogeochemical cycle in aquatic ecosystems. However, how trophic interactions (i.e., biotic factors) regulate the diversity and composition of bacterial community in lake ecosystems remains unknown. Here, we employed DNA meta-barcoding of water samples to explore the impact of bacterivorous protozoans on the bacterial community. The results showed significant seasonal variations in the diversity and composition of both bacterial and protist communities. The composition of bacterivorous protozoans was identified as the primary predictor for the bacterial community alpha diversity in spring and summer, and for beta diversity in spring and autumn, indicating that biotic interactions play a greater role in driving the diversity of bacterial community across different seasons. Biological factors were more important than environmental factors for explaining the variations in the relative abundance of several bacterial genera (i.e., *Pseudoxanthomonas*, *hgcI_clade*, and *Pseudorhodobacter*). Network analyses showed that bacterial networks differed among seasons, and the autumn network exhibited the highest stability. Our findings indicated that the bacterial community stability was significantly affected by environmental factors, specifically SO_4_^2–^and PO_4_^3–^, rather than bacterivorous protozoans. Overall, our findings provide new perspectives on the role of trophic interactions in maintaining the structure of bacterial community in different seasons, and enhance our understanding of the bacterial community assembly in lake ecosystems.

**Supplementary Information:**

The online version contains supplementary material available at 10.1007/s42995-024-00256-8.

## Introduction

Microorganisms are integral components of lake ecosystems and play crucial roles in biogeochemical cycles (nutrient cycling and self-purification) and the overall health and productivity of the planet (Gralka et al. 2020; Li et al. 2023). Microbes are highly diverse and are essential for maintaining ecosystem stability and functions (Li et al. 2021). Despite their critical roles, it remains uncertain how the microbial communities assemble (i.e., what drives their composition and diversity) and how they will respond to future environmental changes.

In recent years, the structure of microbial communities as well as the factors influencing them have been studied in freshwater ecosystems (Humbert et al. 2009; Shang et al. 2022; Wu et al. 2007). For example, the entire bacterial community exhibited the seasonal succession and spatial distribution in lake (Zhu et al. 2019). Significant seasonal differences were observed in the α diversity and composition of microorganisms in lake (Shang et al. 2022). Seasonal variation had a stronger impact on bacterial community structure than any other variation in the river (Yang et al. 2022). The composition and functions of microbial communities greatly vary during different seasons (Yadav et al. 2021). In addition, the composition and diversity of microorganisms in water play a significant role in the microecological functions of lakes (Abdullah AI et al. 2022; Chen et al. 2023; Kritzberg and Bååth 2022). Therefore, it is crucial to elucidate the mechanisms underlying the composition, diversity, and functional structure of microbial communities to understand the structure and function of lake ecosystems.

To date, the majority of research on the impact of bacterial community has focused on abiotic factors (Shang et al. 2022), for example, environmental factors (pH and temperature), nutrient in the water (total carbon and nitrogen), and climate conditions can influence the bacterial community structure (Abdullah Al et al. 2022; Zou et al. 2021). However, the importance of biotic interactions to the structure of bacterial community has been overlooked (Sun et al. 2022). Indeed, biotic interactions (e.g., predation, competition, and mutualism) are important for nutrient and vitamin exchange through microbial food webs (Li et al. 2023; Logares et al. 2020; Pernthaler 2005). Protozoa have been shown to prey mainly on bacteria and fungi (Geisen 2016), and the activity of protozoa can affect community assembly through top-down processes (Nguyen et al. 2021). Several studies have indicated that protists grazing can selectively reduce the relative abundance of some bacteria groups (i.e., unicellular cyanobacteria or certain betaproteobacteria) (Pernthaler 2005). Protistan predation affects the evenness, composition, and diversity of bacterial community in microcosms (Batani et al. 2016; Bonkowski 2004; Kuppardt et al. 2018). However, how trophic interactions affect the diversity and composition of bacterial community in aquatic ecosystems remains to be systematically understood.

Network approaches are often used to explore the interactions between microbes and their responses to environmental changes (Liu et al. 2022; Yuan et al. 2021). A central yet strongly debated concern centers on the factors that affect the complexity and stability of a community, and whether and how ecosystem stability is influenced by the network complexity (Hillebrand et al. 2018; Yuan et al. 2021). Several studies have indicated that the complexity and stability of bacterial networks can be enhanced by climate change (Yuan et al. 2021) and that bacterial stability is also regulated by keystone taxa (Liu et al. 2022). Moreover, microbial interactions play critical roles in stabilizing network structures in different natural environments (Li et al. 2023; Sun et al. 2022). However, it remains uncertain whether and how microbial interactions affect bacterial community stability.

Most alpine lakes are relatively closed ecosystems and are particularly sensitive to environmental changes and human activities (Liu et al. 2023; Obertegger and Flaim 2021). Such lakes thus provide ideal natural experimental sites for investigating the diversity and stability of microbial communities in different seasons. In our study, we used 16S and 18S ribosomal RNA (rRNA) gene sequencing to examine bacterial and protist communities sampled from the center of a subalpine lake. The aims were (i) to determine the relative contribution of environmental and biological factors in maintaining the diversity and composition of bacterial community, (ii) to uncover the factors that best explain the variation in bacterial community stability.

## Materials and methods

### Study sites and sampling

Gonghai Lake (38.91°N, 112.23°E, altitude 1854 m) is located in Ningwu County, Shanxi Province, China (Supplementary Fig. S1). It is characterized by an annual average temperature of 6.2 °C and an average annual rainfall of 490 mm (Liu et al. 2021). The lake is a small enclosed freshwater lake and precipitation is the main source of water, with an elevation of 1854 m and a maximum depth of approximately 8 m. Water samples were collected every 3 m from top to bottom using a Plexiglass water sampler (LB-800, Qingdao, China) at the center of the lake during spring (May 9th), summer (August 6th), and autumn (October 11th) in 2021 and winter (January 8th) in 2022, respectively. Water was sampled three times at each sampling point (0 m, 3 m, and 6 m) (Supplementary Fig. S1). A total of 36 water samples were collected. Then, approximately 2.5 L of the water from each sampling site was filtered through a 0.22 μm polycarbonate membrane (Millipore, Jinteng, Tianjin, China), and the membrane was stored at – 80 °C until DNA extraction. The physicochemical properties of the water (e.g., pH and sulfate content) were measured as described by Liu et al. (2021) (Supplementary Table S1).

### DNA extraction and sequencing

The FastDNA® SPIN Kit (MP Biomedicals, USA) was employed to obtain water DNA from the filter membranes. The protist 18S rRNA V9 region and bacterial 16S rRNA V3–V4 region were amplified using the primers 338F/806R and TAReuk454F/TAReuk454R (Inaba et al. 2020; Yao et al. 2019). The purified PCR products were sequenced using an Illumina MiSeq platform (Shanghai, China). The bacteria and protists were assigned by SILVA (v138.1) and PR2 (v.4.14) (Guillou et al. 2013; Quast et al. 2013). We removed sequencing reads of fungi, metazoans, archaea, and unclassified sequences. Potential functions of bacterial community were identified by the functional annotation of prokaryotic taxa (FAPROTAX) (Abdullah Al et al. 2022; Hu et al. 2022), including involvement in carbon, sulfur, and nitrogen cycles, and pathogens. Protists are unicellular and multicellular algae and protozoans with a wide range of ecological functions (Gran-Stadniczenko, 2019). The protist functional groups, including algivores, bacterivores, mycophages, nonselective omnivores, heterotrophic parasites, phototrophs, raptors, saprotrophs, and unknown, were classified according to previously described feeding modes (Bjorbækmo et al. 2020; Li et al. 2022; Sun et al. 2021; Wu et al. 2022; Xiong et al. 2018, 2021) (Supplementary Table S2).

### Statistical analysis

#### Driving factors of bacterial community structure

Microbial diversity (richness and Shannon index) was calculated using the “vegan” package in R (v. 4.1.3) (Oksanen et al. 2017). The variation in community composition was evaluated by PCoA. The significant differences between seasons were determined by ANOSIM.

The richness (OTUs), Shannon diversity, and composition (two beta-PCoA axes) of bacterivorous protozoans represented biotic factors. To examine the effects of abiotic and biotic variations on bacterial alpha diversity and composition, we employed random forest analysis (“rfPermute” package) (Archer 2013) and a multiple regression model (“stats” package) (Field, 2012; Grömping 2006). Mantel and partial Mantel tests (“ecodist” package) were used to assess the effects of all variations on community structure (Goslee, 2007). We applied db-RDA to evaluate the major factors driving bacterial beta diversity (Oksanen et al. 2015).

### Network construction and stability analysis

To explore potential interactions among the four seasons, a co-occurrence network between bacterial and protist taxa was constructed using Spearman’s correlation coefficients (|r|> 0.6, *p* < 0.05) and was visualized in Gephi (version 0.9.2) (Csárdi et al. 2006; Delgado-Baquerizo et al. 2020). In addition, we constructed a single-kingdom network. To understand whether biotic factors (absence or presence of bacterivorous protozoans) influence the bacterial community stability, indices were used to reflect networks stability, such as robustness (Dunne et al. 2002; Montesinos-Navarro et al. 2017), vulnerability (Herren and McMahon 2017), cohesion (Liu et al. 2022), and natural connectivity (Peng and Wu 2016). We constructed PLS‒PM using the R package “plspm” to verify the impacts of all variables on community stability (Gao et al. 2019; Liu et al. 2022).

## Results

### Seasonal dynamics of bacterial and protist communities

**Fig. 1:**
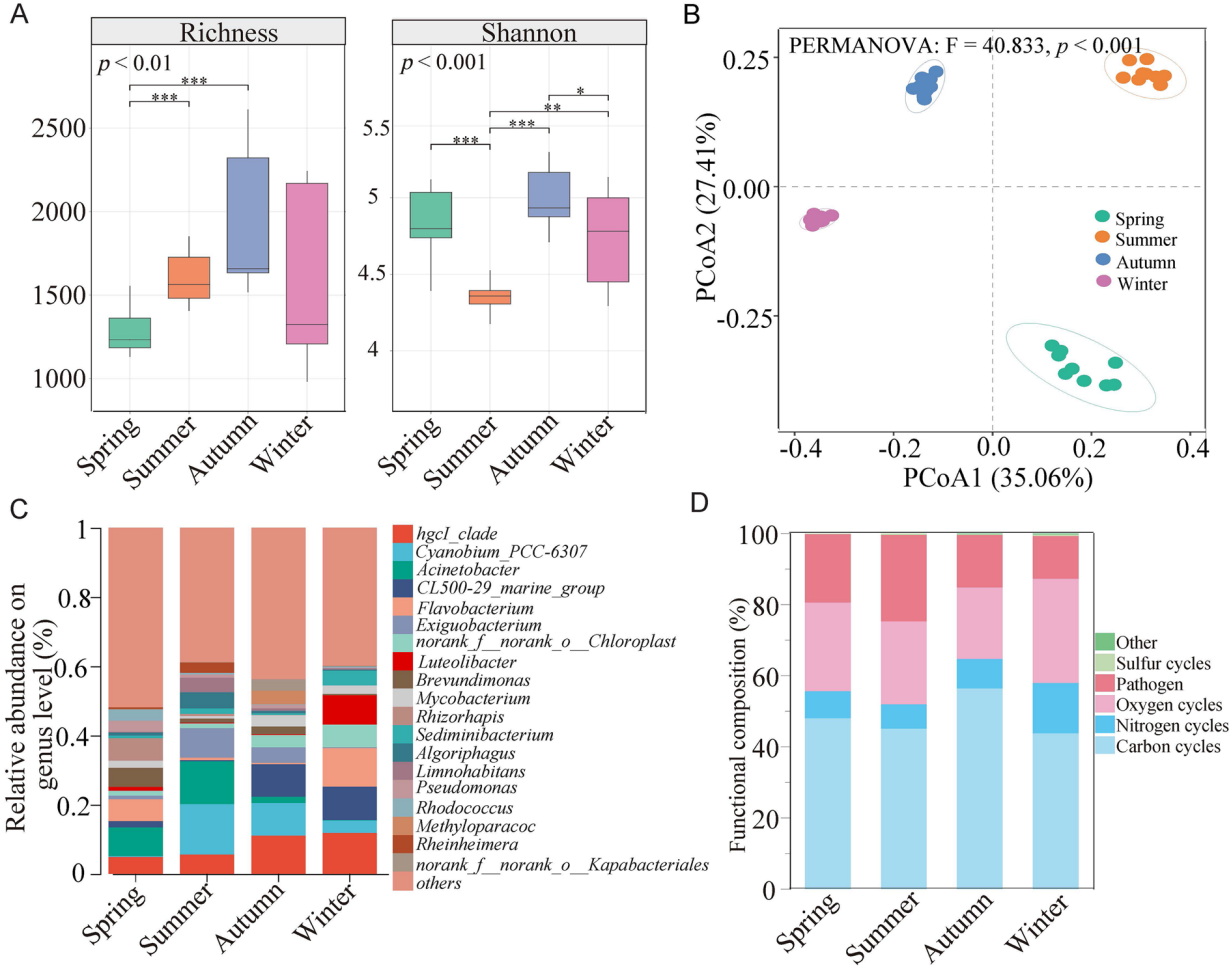
Diversity, composition, and abundance of bacteria in different seasons. **A** Bacterial alpha diversity. **B** Principal coordinate analysis (PCoA) of the seasonal dynamics of bacterial community. **C** Relative abundance of bacteria at the genus level. **D** Functional composition of bacterial community

A total of 6,959 OTUs (3,985,776 sequences) for bacteria and 953 OTUs (152,928 sequences) for protists were obtained in the survey area. The bacterial richness was markedly higher (*p* < 0.05) in summer than in spring, while the Shannon index in summer was lower than that in winter (Fig. 1A). The Shannon index of protist community was higher (*p* < 0.05) in spring than that in winter, while the richness showed no significant changes across spring and summer (*p* > 0.05) (Supplementary Fig. S2A). The principal coordinate analysis (PCoA) of Bray‒Curtis distances revealed that the beta diversities of both bacterial and protist communities exhibited distinct differences (analysis of similarities ANOSIM, *p* < 0.05) and were clustered into four groups according to seasonal changes (Fig. 1B, Supplementary Fig. S2B). The bacterial community was dominated by *hgcI_clade* (8.36%), *Cyanobium_PCC-6307*(6.94%), *Acinetobacter* (5.66%), *CL500-29_marine_group* (5.31%), *Flavobacterium* (4.64%) and *Exiguobacterium* (3.58%) (Fig. 1C). The most abundant genera in the protist communities were *Ceratium* (5.71%), *Botryococcus* (5.00%), *Choricystis* (4.54%) and *Stephanodiscus* (4.51%) (Supplementary Fig. S2C). These dominant genera of the bacterial and protist communities displayed significant (*p* < 0.05) seasonal changes in abundance (Supplementary Fig. S3). The bacterial functional groups involved in carbon cycles comprised the largest proportion during the four seasons, followed by those involved in oxygen cycles and then pathogens (Fig. 1D). The functional groups involved in carbon cycles were relatively more abundant in autumn, while those involved in nitrogen and oxygen cycles were increasingly abundant in winter (Fig. 1D). The protists were functionally classified as algivores, bacterivores, parasites, mycophages, nonselective omnivores, phototrophs, raptors, saprotrophs, and unknown, where the phototrophs and bacterivores were the dominant groups in all seasons, except for unknown groups (Supplementary Fig. S2D).

To unravel the potential biological interactions between bacterial and protistan communities, a co-occurrence network was constructed (Supplementary Fig. S4). The network consisted of 264 nodes and 5078 edges, with 55.84% positive and 44.16% negative correlations. Among the connections, 709 were found between bacterial taxa and protistan taxa (Supplementary Fig. S4). Notably, the protistan functional groups in protist–bacterial interactions were dominated by phototrophs (245 connections), including Chlorophyta, Cryptophyta, and Ochrophyta, followed by bacterivores (133 connections) including Ciliophora, Ochrophyta, Lobosa, and Cercozoa. Phototrophs and bacterivores as the dominant aquatic protistan functional groups (Supplementary Fig. S2D), had the strongest associations with bacterial community (Supplementary Fig. S4). Given these results, we focused subsequent analyses on bacterial and bacterivorous protozoan communities in different seasons.

### Drivers maintaining the diversity and composition of bacterial community

**Fig. 2:**
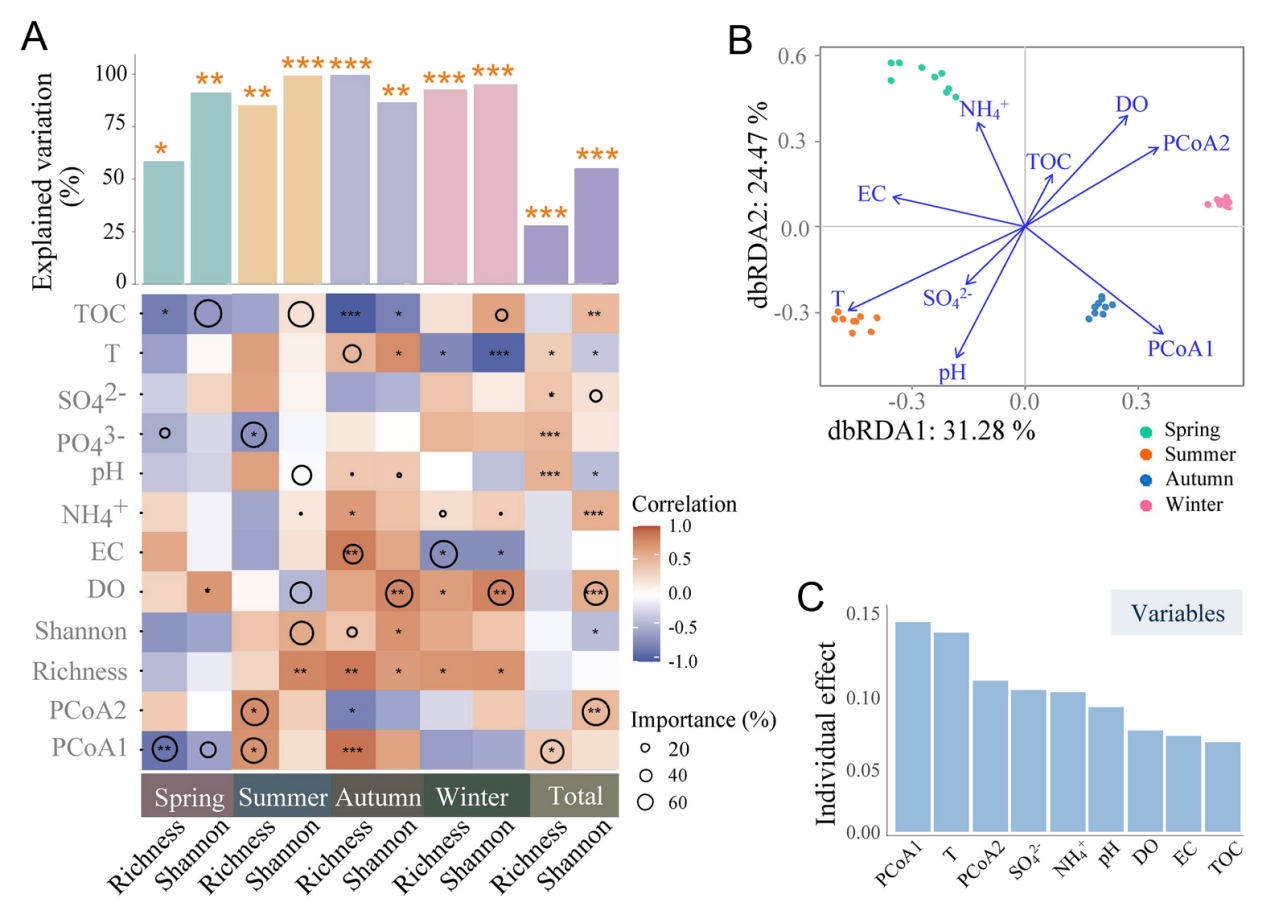
Driving factors of bacterial diversity in different seasons. **A** The relative importance of different variables to bacterial alpha diversity; importance is represented by circle size. The color represents the strength of the Spearman correlation. **B** Distance-based redundancy analysis (db-RDA) indicating different factors that affected bacterial beta diversity. **C** Individual effects of different factors on bacterial community. Richness: operational taxonomic unit (OTU) richness of the bacterivorous protozoans community; Shannon: the OTU Shannon index of the bacterivorous protozoans community; Composition: PCoA1 and PCoA1 of the bacterivorous protozoans community

Given that the composition and alpha diversity of bacterial community exhibit seasonal dynamics, we investigated the biotic and abiotic factors driving bacterial community structure. Bacterivorous protistans were dominant during the four seasons (Supplementary Fig. S2D) and had strong associations with bacterial taxa (Supplementary Fig. S4). Therefore, the diversity (richness and Shannon index) and composition (PCoA1 and PCoA2) of bacterivorous protozoans community were used to infer the biotic factors. The composition of bacterivorous protozoans community significantly related to bacterial alpha diversity (Fig. 2A), as assessed by random forest analysis and multiple stepwise regression model analysis. This result was identified via positive simple linear regressions, and a significant correlation was found between bacterivorous protozoans composition (PCoA1 and PCoA2) and bacterial diversity (Supplementary Fig. S5). Only the bacterivorous protozoans composition significantly explained the bacterial richness in spring and summer, and it did not explain the bacterial diversity in autumn and winter (Fig. 2A).

In terms of seasons, Mantel and partial Mantel tests indicated that biotic variables were more important predictors of bacterial community dissimilarity than abiotic variables (Supplementary Table S3). The influence of biotic factors was strong in summer and winter (*r* = 0.82 and 0.84, *p* < 0.001) but weak in spring and autumn. Distance-based redundancy analysis (db-RDA) revealed that temperature and PCoA1 of bacterivorous protozoans community were the dominant predictors (Fig. 2B), and PCoA1 had the greatest individual effect on bacterial beta diversity (Fig. 2C). The dissimilarity of bacterivorous protozoans community was significantly linked to the dissimilarity of bacterial community (Supplementary Fig. S5). Our analysis revealed that the composition of bacterivorous protozoans affected bacterial diversity.

**Fig. 3:**
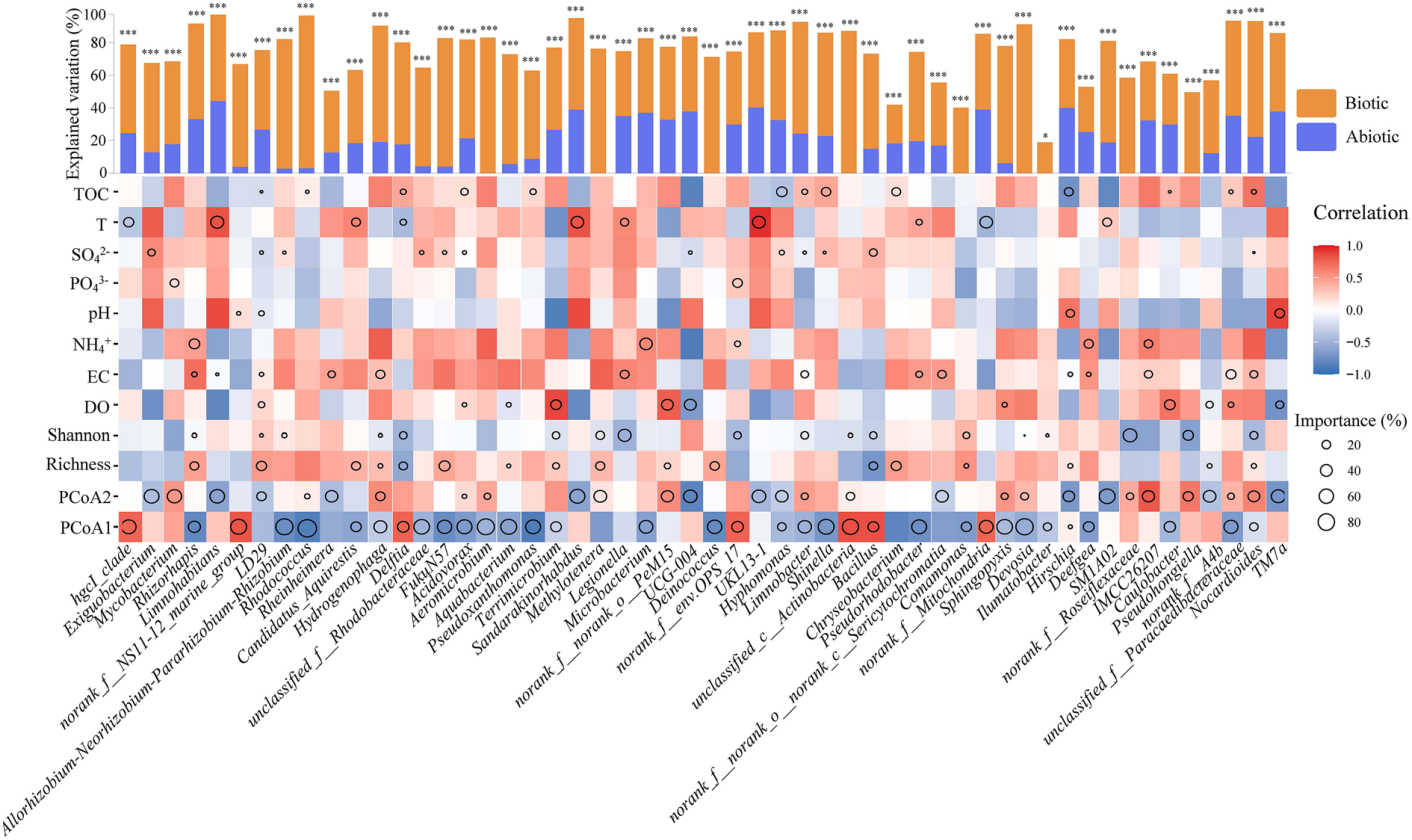
Potential contributions of biotic and abiotic factors to the abundance of the bacterial genera (the top 10% in terms of relative abundance). The circle size represents the variable importance. The color represents the strength of the Spearman correlation

We further analyzed the relative importance of all variables in explaining the variables in relative abundance of major genera by using multiple regression models and variance decomposition (Fig. 3). We found that the composition and diversity of bacterivorous protozoans had a stronger effect on 53 bacterial genera, including *Pseudoxanthomonas*, *Comamonas*, and *Methylotenera* (Gammaproteobacteria), *hgcI_clade*, *Rhodococcus*, and *Ilumatobacter* (Actinobacteria), *Caulobacter*, *Hyphomonas*, and *Pseudorhodobacter* (Alphaproteobacteria), and *Chryseobacterium* and *norank_f__NS11-12_marine_group* (Bacteroidia). Thus, our results revealed that potential trophic interactions existed between specific bacterial genera and bacterivorous protozoans. Furthermore, the relative abundances of bacterivorous protozoans and specific bacteria in spring were greater than those in other seasons (Supplementary Fig. S6A and B). The relative abundance of bacterivorous protozoans increased with the specific bacterial abundance during the four seasons (Supplementary Fig. S7A). The trophic transfer efficiency in microbial communities was estimated by the ratio of bacterivorous protozoans predators to specific bacteria. This finding indicated that the ratio was higher in autumn (Supplementary Fig. S7A). However, the relative abundance of bacterivorous protozoans predators and specific bacteria had the strongest positive effect in summer (*R* = 0.76, *p* < 0.05), rather than in the other seasons (*p* > 0.05) (Supplementary Fig. S7B). Our data suggested that trophic interaction was the major driver for specific members of the bacterial community.

### Factors underlying the stability of bacterial community

**Fig. 4:**
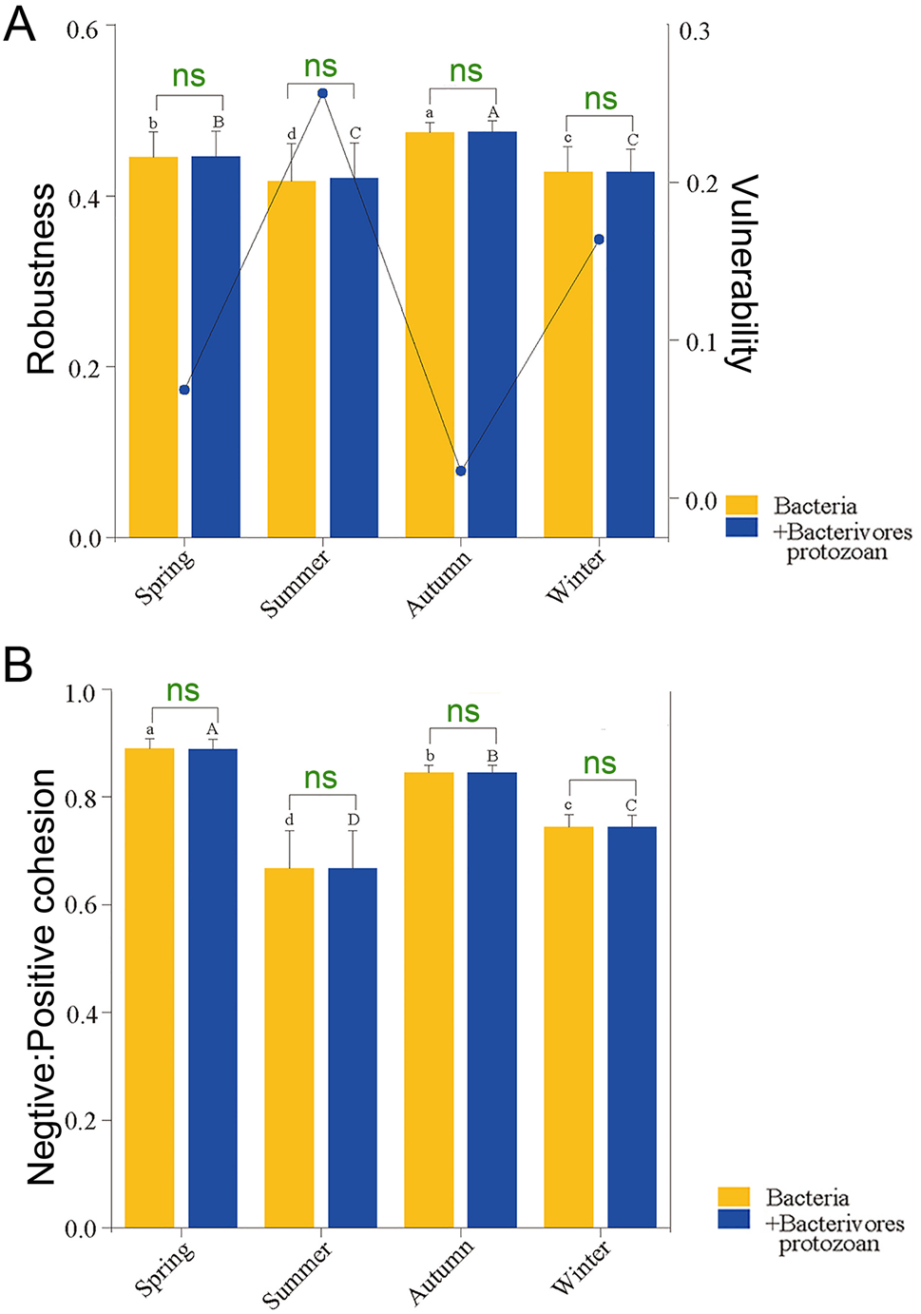
Network stability in different seasons. **A** Robustness and vulnerability **B** Negative: positive cohesion of different networks. Different letters indicate significant differences in different seasons (one-way ANOVA, *p* < 0.05)

**Fig. 5:**
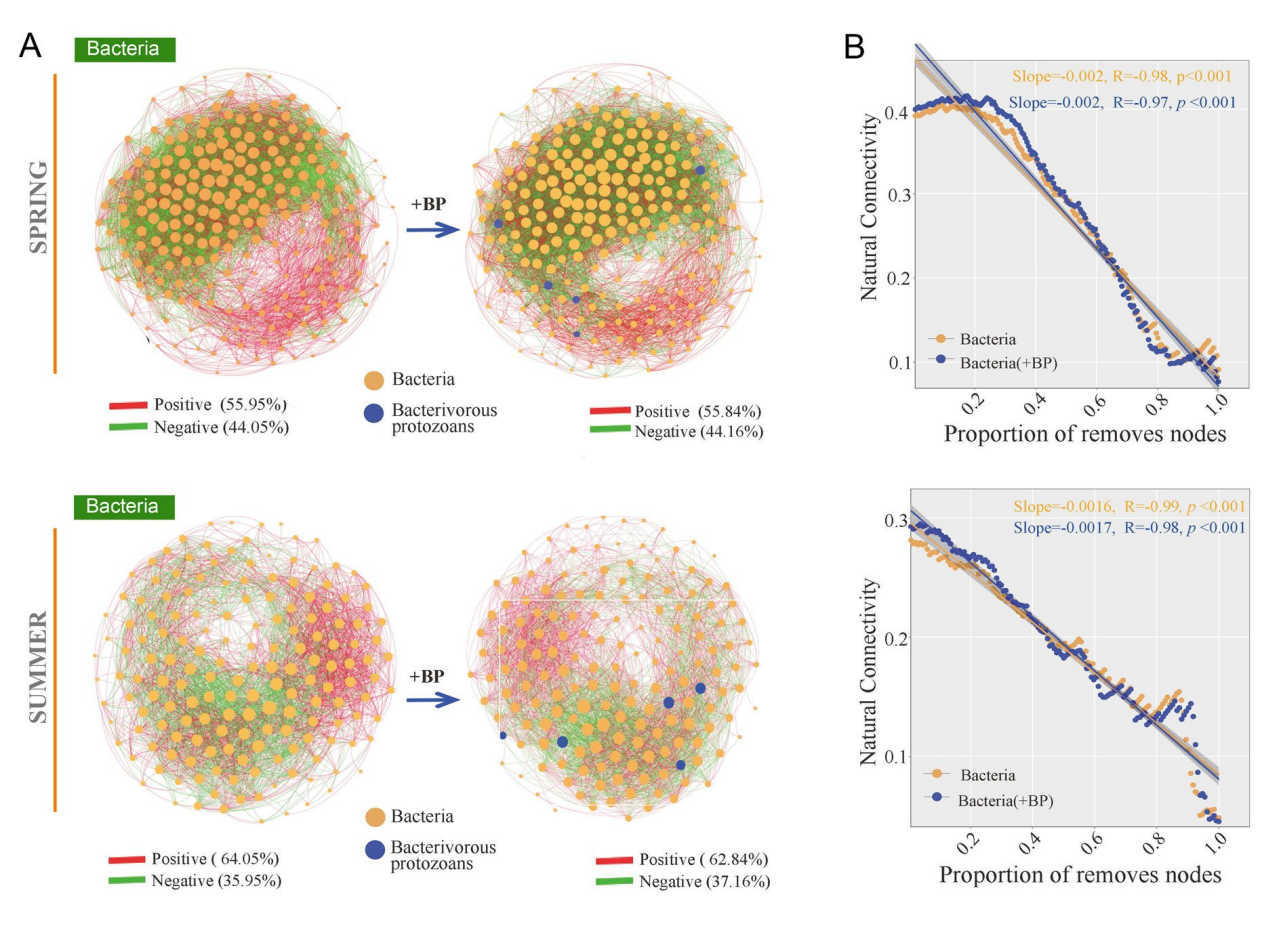
The co-occurrence network of bacterial community with the addition of bacterivorous protozoans in spring and summer. **A** Changes in network topology with bacterivorous protozoans addition. **B** Changes in network stability with bacterivorous protozoans addition. The R-values and slopes are shown in the diagrams. “ + BP” represents the addition of bacterivorous protozoans relationships to the bacterial community

**Fig. 6:**
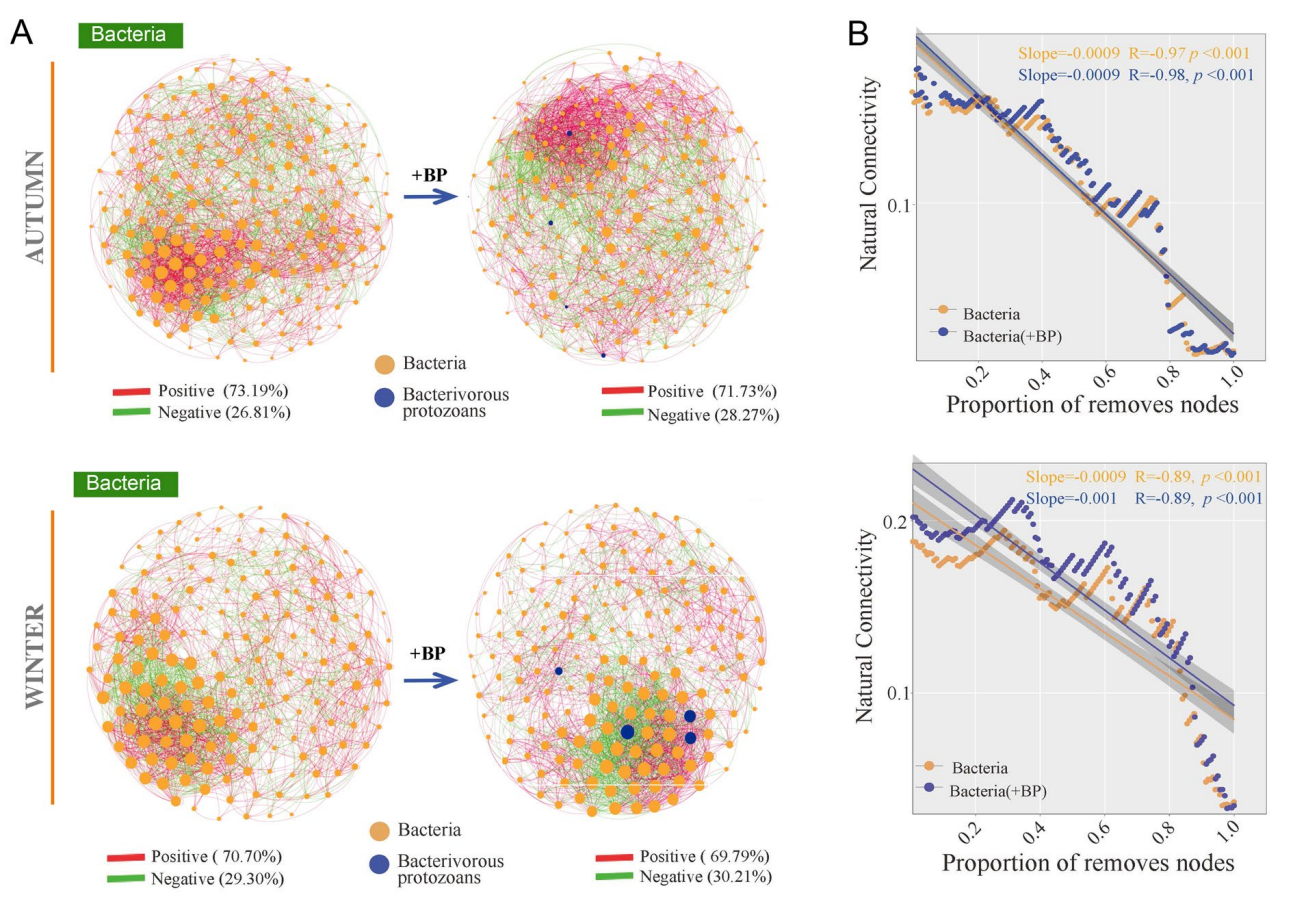
The effect of bacterivorous protozoans interactions on networks of bacteria in autumn and winter. **A** Changes in network topology resulting from the addition of bacterivorous protozoans interactions. **B** The impact of bacterivorous protozoans interactions on network stability

With the addition of bacterivorous protozoans, the average degree, network density, network modularity, and average clustering coefficient increased gradually in autumn, while the nodes and edges of the networks did not change (Supplementary Fig. S8). These results suggested that the network in autumn exhibited higher complexity in the presence of bacterivorous protozoans. The network stability was evaluated by using robustness, vulnerability, and cohesion indices. Based on the presence or absence of bacterivorous protozoans, the robustness results confirmed that the autumn network (0.474 ± 0.002) had a markedly higher robustness than the other seasons (*p* < 0.05) (Fig. 4A). The network vulnerability in autumn was more stable than those in spring, summer, and winter (Fig. 4A). The spring (0.889 ± 0.003) and autumn (0.845 ± 0.013) networks had higher negative:positive cohesion values than the summer (0.668 ± 0.012) and winter (0.744 ± 0.004) networks (Fig. 4B). The natural connectivity of bacterial networks was more stable in autumn (slope = − 0.0009) and winter (slope = − 0.0009), whereas the slopes were unchanged (slope = − 0.0009 and − 0.001), with bacterivorous protozoans added to the network (Fig. 5, 6). However, the robustness, vulnerability, negative:positive cohesion, and natural connectivity of networks showed no significant difference between the single-kingdom bacterial network with and without bacterivorous protozoans addition (Fig. 4, 5, 6, Supplementary Fig. S9). These results indicated that the autumn network was the most stable, while there was no significant difference in network stability following the addition of bacterivorous protozoans to the network.

**Fig. 7:**
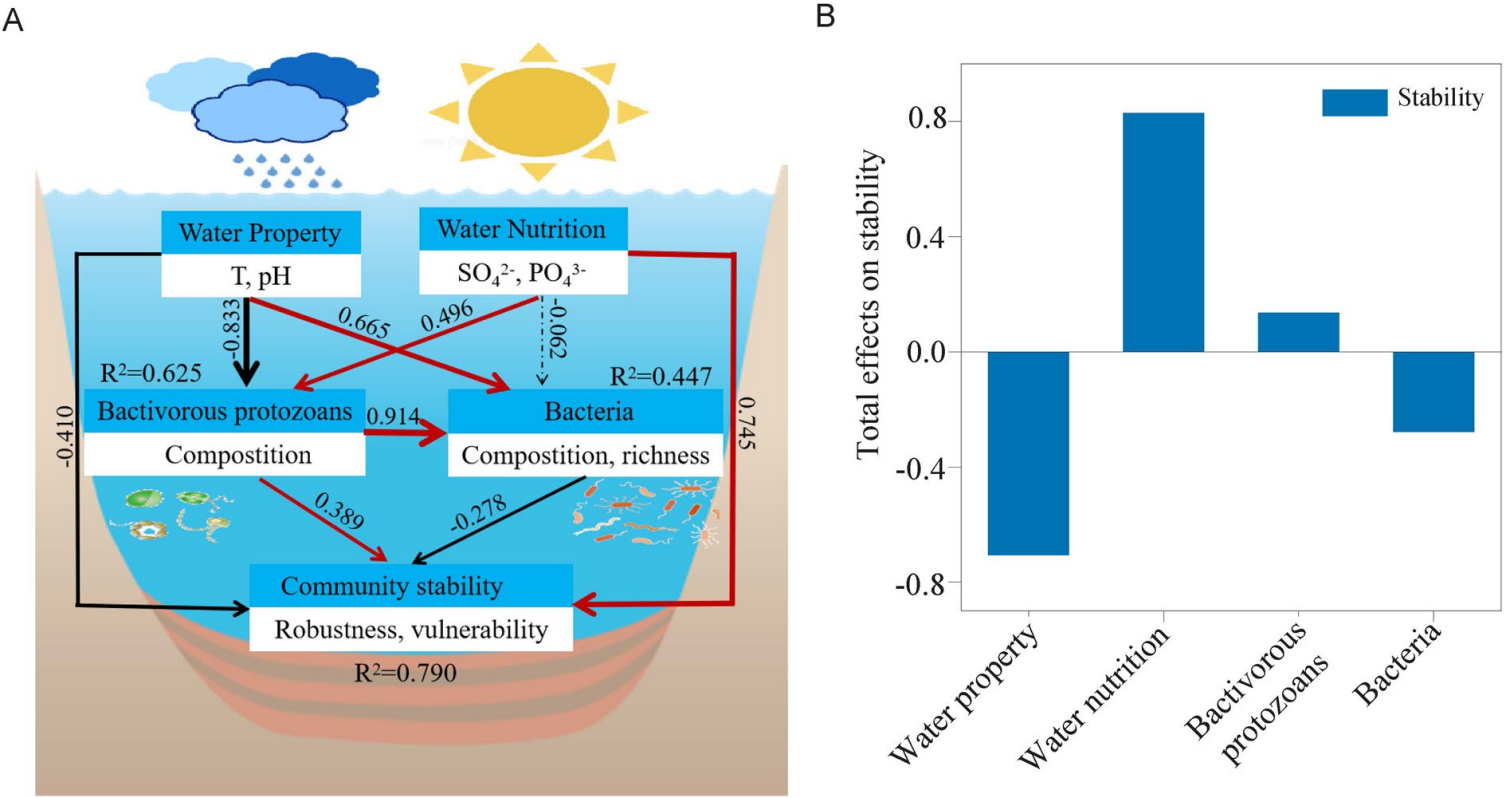
Contribution of abiotic and biotic factors to bacterial community stability. **A** Partial least-squares path model (PLS-PM) shows the direct and indirect effects. The width of the arrows is proportional to the strength of the path coefficients. Red and black lines represent positive and negative adjusted path coefficients, respectively. Continuous and dashed arrows indicate significant and nonsignificant relationships, respectively. *R*^2^ denotes the proportion of the variance explained. **B** Standard effects of the latent variables on bacterial community complexity and stability

A partial least-squares path model (PLS-PM) (GOF value = 0.653, Fig. 7A) was constructed to further analyze the underlying mechanism of bacterial community stability in freshwater ecosystems. Our results indicated that bacterivorous protozoans components were the most important contributors influencing bacterial diversity and composition (path coefficient = 0.914, *p* < 0.001). Bacterivorous protozoans were positively associated with bacterial community stability, while bacterial diversity and composition displayed significantly negative relationships with community stability (Fig. 7A). Moreover, the aquatic nutrient content (path coefficient = 0.745, *p* < 0.001) was the major driver affecting the stability of bacterial community. Thus, these data suggested that environmental factors (SO_4_^2–^ and PO_4_^3–^) were more effective in influencing community stability (Fig. 7).

## Discussion

### Influence of biological factors on the diversity and composition of bacterial community

Our data suggested that the composition of bacterivorous protozoans was one of the primary predictors of bacterial diversity compared with water physicochemical properties (Fig. 2), indicating a major role of biotic interactions in shaping the bacterial community diversity. A previous study also indicated that predatory protists strongly influenced bacterial diversity and productivity (Pernthaler 2005). For example, the feeding of heterotrophic dinoflagellates affects bacterial species richness and alters energy flow processes in marine environments (Saleem et al. 2012). Predation by ciliates (e.g., *Metopus* and *Caenomorpha*) can regulate the structure and function of bacterial community in anoxic ecosystems (Hirakata et al. 2016). Specifically, the composition of bacterivorous protozoans was the most important variable for explaining bacterial richness in spring and summer, while biotic factors showed a strong correlation in summer and winter (Supplementary Table S3). Possible reasons are that bacterivorous protozoans have different prey preferences, suggesting that selective grazing influences the structure of bacterial community (Bunse and Pinhassi 2017).

Our results also confirmed that biotic factors explain more variations of specific members in bacterial community (Fig. 3). Bacterial grazers have different feeding modes, such as filtering, direct interception, and raptorial modes, which may influence their effects on the bacterial community (Bunse and Pinhassi 2017; Jeuck and Arndt 2013). Some bacterivorous protozoa (e.g., Telonemia) and algae are essential for community assembly or bacterial diversity (Klaveness et al. 2005; Li et al. 2023). Compared with other seasons, the relative abundance of bacterivorous protozoans and specific bacteria was higher in spring (Supplementary Fig. S6A and B). Our result was supported by previous research showing that dissolved organic matter released by phytoplankton decay increased bacterial production and growth after the spring bloom (Bunse and Pinhassi 2017). A vernal alga and bacterial bloom provides ciliates and heterotrophic nanoflagellates with rich food sources (Posch et al. 2022). We also noted that the trophic transfer efficiency was greater in autumn (Supplementary Fig. S7A), which may be related to the presence of carbon cycle-associated bacteria, which were more numerous in autumn (Fig. 1D). For example, Gammaproteobacteria was the most dominant group during the four seasons, and was essential for carbon and nitrogen cycling (Newton et al. 2011; Shang et al. 2022). Additionally, protists mainly graze on Cyanobacteria, Gammaproteobacteria, and Bacteroidetes (Pernthaler 2005). We also noted an association between the relative abundance of protozoans grazing on Gammaproteobacteria and Gammaproteobacteria, and the ratio was higher in autumn (Supplementary Fig. S6C and D). Overall, our results suggested that biotic factors can regulate bacterial community diversity and composition and may potentially affect nutrient cycling in aquatic ecosystems.

### Factors affecting the stability of bacterial community

Our data indicated that the networks of bacterial community were distinct across seasons, with autumn exhibiting the highest stability (Fig. 4). When bacterivorous protozoans were integrated into the bacterial networks, the stability of the network was not enhanced in different seasons (Figs. 5, 6). However, the results of previous studies are not consistent in stating that biotic interactions are essential for maintaining bacterial community stability (Liu et al. 2022). One possible reason for this is that these biotic interactions in the co-occurrence network are only putative species interactions. Other studies have reported that in systems where protistans graze on bacteria, the bacteria can resist predation by forming filaments or colonies through oversizing, miniaturizing, forming biofilms, practicing allelopathy, or using other inducible defense mechanisms (Bertilsson and Mehrshad 2022). These defense mechanisms can impact bacterial community dynamics, diversity, and composition. In addition, predatory protists can transfer organic carbon to higher trophic levels through microbial food webs, and predatory action can stabilize bacterial community by preventing rapid population division (Bertilsson and Mehrshad 2022; Pernthaler 2017). We also noted that SO_4_^2–^ and PO_4_^3–^ significantly affected the stability of bacterial community (Fig. 7). A possible reason for this result is that a subalpine lake can respond more sensitively to changes in the environment, whereas the diversity and stability of bacterial community could be driven by seasonal changes (Guo et al. 2020; Liu et al. 2022). Therefore, the structural dynamics of bacterial community in lakes may be influenced by various factors, ultimately impacting the stability of their network.

## Conclusion

Our data suggested significant seasonal variations in the composition and diversity of bacterial and protist communities. Specifically, the diversity and composition of bacterial community was one of the primarily affected by bacterivorous protozoans composition. Bacterial community stability was significantly affected by environmental factors (i.e., SO_4_^2−^ and PO_4_^3−^) rather than bacterivorous protozoans. To further comprehend the significance of trophic interactions, it is imperative to conduct empirical and theoretical research to elucidate how microbial interactions maintain the mechanisms of microbial diversity and structure in a subalpine lake, particularly in the face of future climate change.

## Supplementary Information

Below is the link to the electronic supplementary material.

Supplementary file1 (DOCX 1839 KB)

Supplementary file2 (XLSX 89 KB)

## Data Availability

The raw sequences of the eukaryotic 18S rRNA and bacterial 16S rRNA genes were deposited in the NCBI GenBank (numbers PRJNA905214 and PRJNA905182).
