# Supplementary material for: Contribution of environmental and biological factors to bacterial community structure and stability in a subalpine lake
Source: Mar Life Sci Technol. 2024 Oct 30;7(1):176–86. doi: 10.1007/s42995-024-00256-8 (PMC11871254; doi:10.1007/s42995-024-00256-8)
Supplement: Supplementary file 1 — Supplementary file1 (DOCX 1839 KB) [file 42995_2024_256_MOESM1_ESM.docx]

**Supplementary materials**


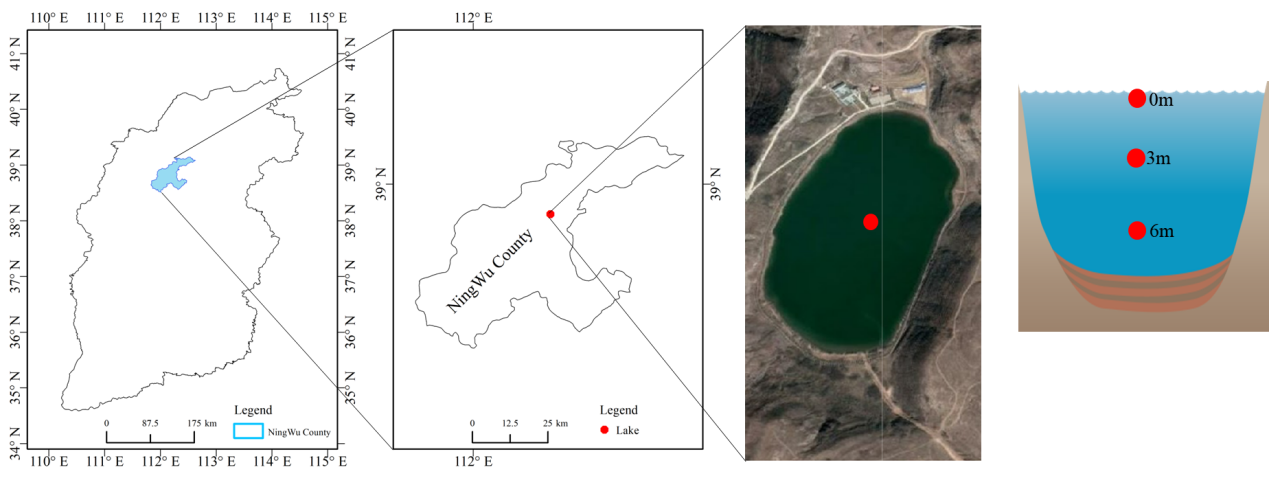


Fig. S1 Map showing the location of sampling sites of Gonghai Lake (GH) in the Ningwu County of Shanxi, China.


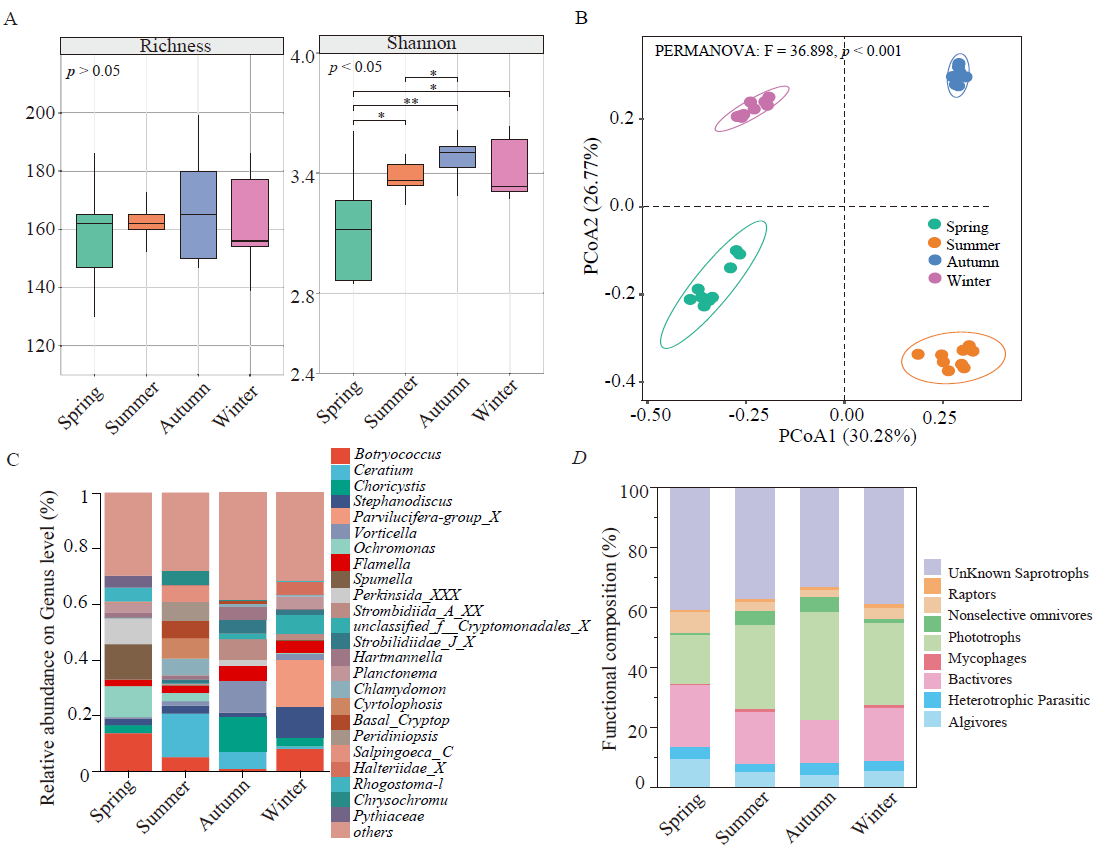


Fig. S2 Diversity, composition and abundance of protists in different seasons (A) Protists alpha diversity (richness and Shannon diversity). (B) Principal coordinate analysis (PCoA) of seasonal dynamics of protists communities. (C) Relative abundance of protists at the genus level. (D) The functional composition of protists communities.


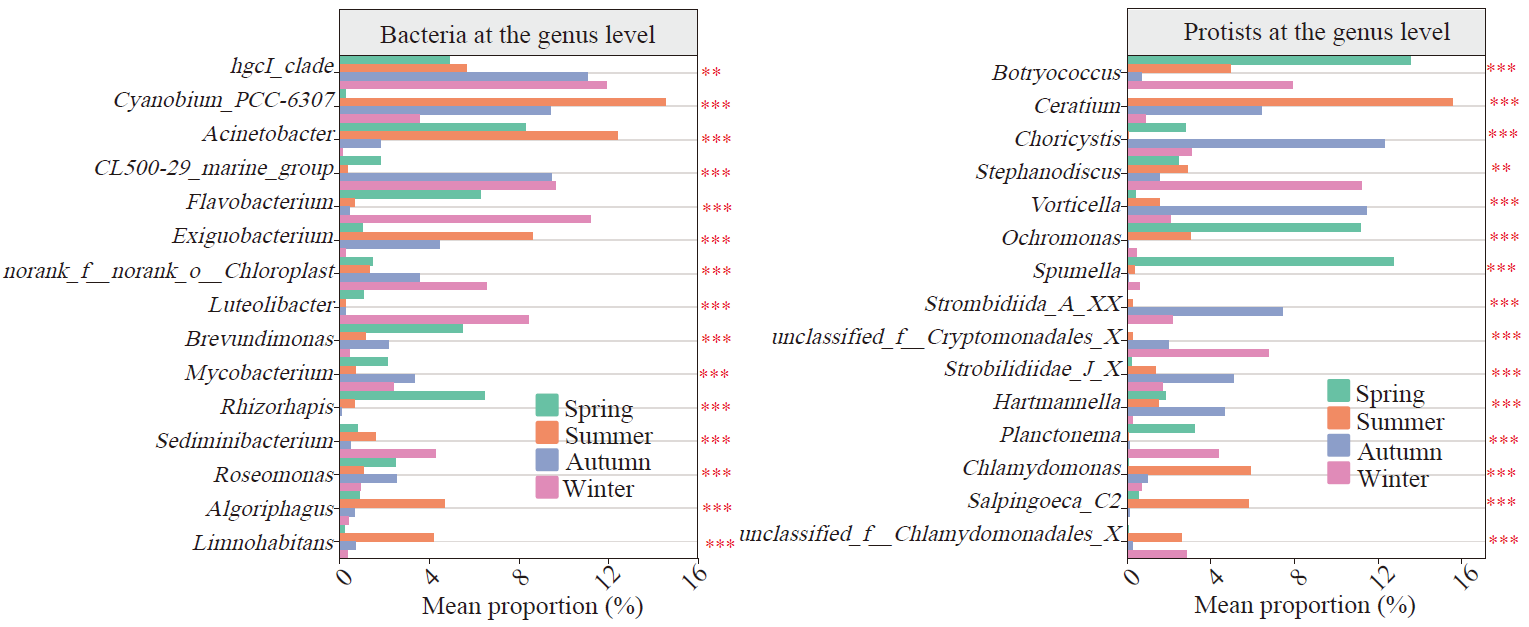


Fig. S3 The variation analysis of dominant genus of bacterial and protists communities in different seasons (*, *p* < 0.05; **, *p* < 0.01; ***, *p* < 0.001).


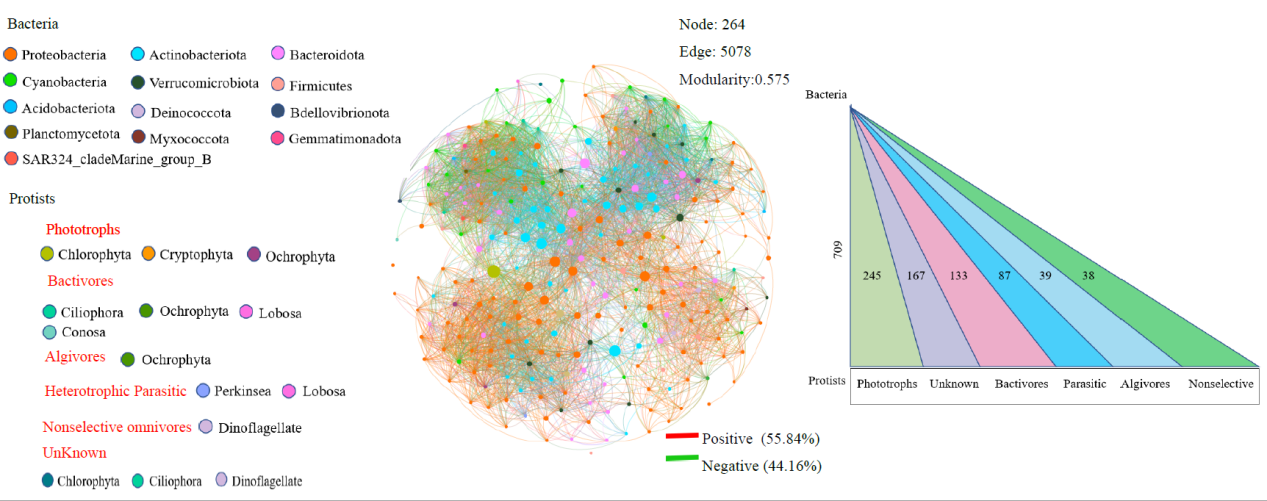


Fig. S4 The co-occurrence network of bacterial and protists communities in different seasons. Edges only show strong (Spearman correlation > |0.6|) and significant (*p* < 0.05) connections. The size of each node is proportional to the number of connections to it. A summary of node-edge statistics is provided at the left of the network.


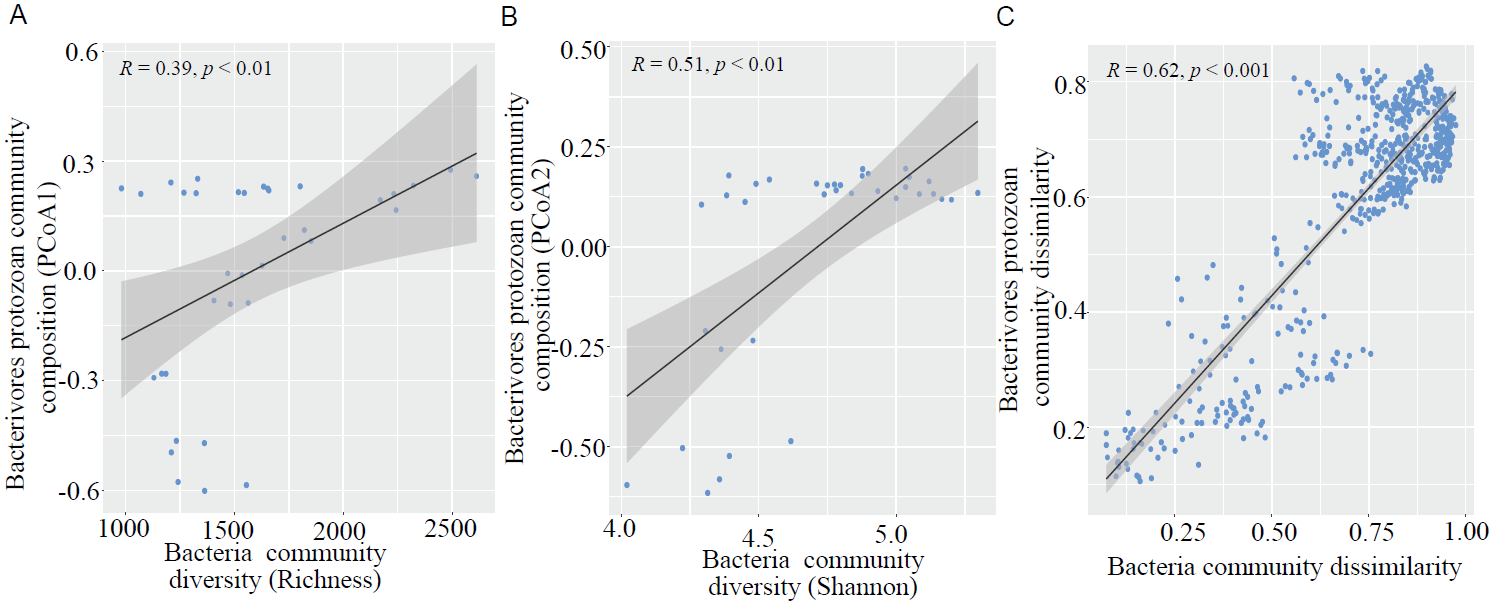


Fig. S5 Relationships between bacteria community diversity and the main drivers were examined by linear least‐squares regression analysis. (A) Relationships between bacteria richness and bacterivorous protozoans composition (PcoA1). (B) Relationships between bacteria Shannon index and bacterivorous protozoans composition (PcoA2). (C) Relationships between bacteria community dissimilarity and bacterivorous protozoans community dissimilarity.


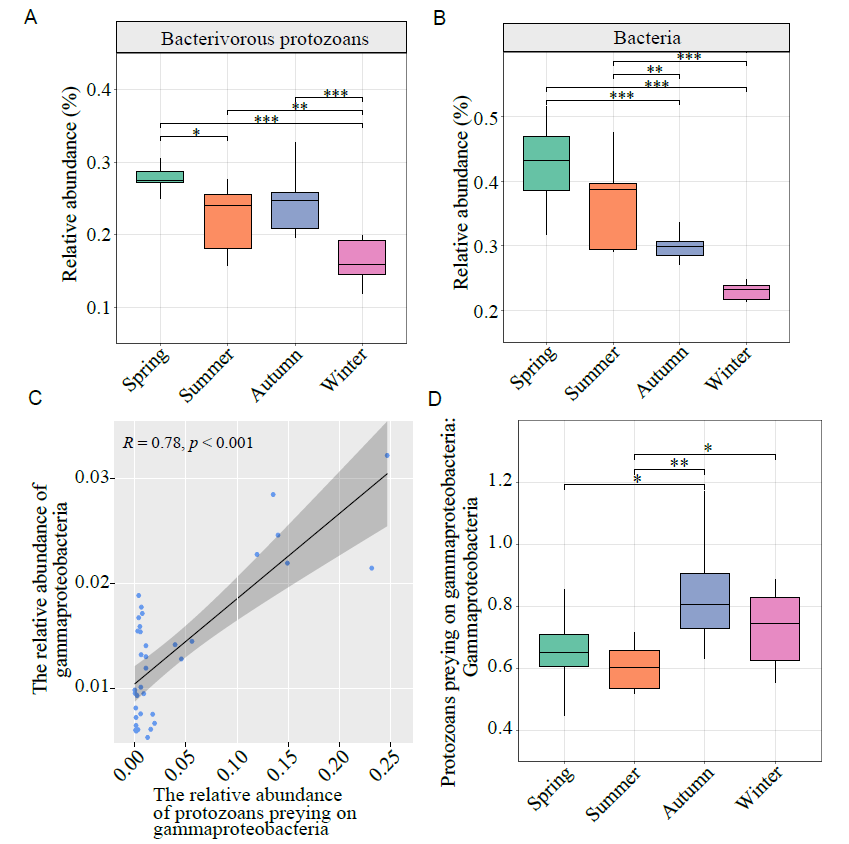


Fig. S6 Relative abundances of bacterivorous protozoans predators (A) and specific bacteria (B). (C) Correlation analysis between protozoans preying on gammaproteobacteria and gammaproteobacteria. (D) Ratio between protozoans preying on gammaproteobacteria and gammaproteobacteria

Protists


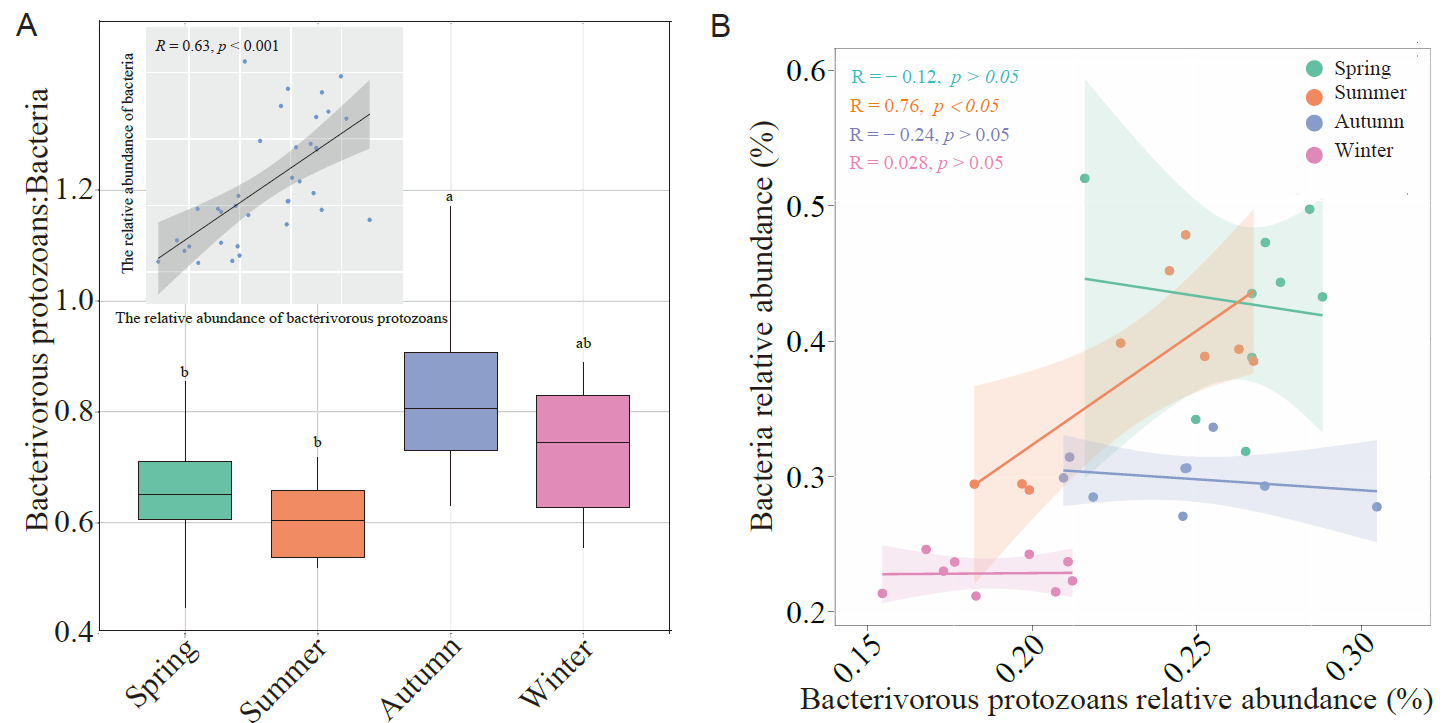


Fig. S7 (A) Correlation analysis and ratio between bacterivores protozoan predators and specific bacteria the whole seasons. (B) Correlation analysis between bacterivores protozoan predators and specific bacteria in spring, summer, autumn, and winter.


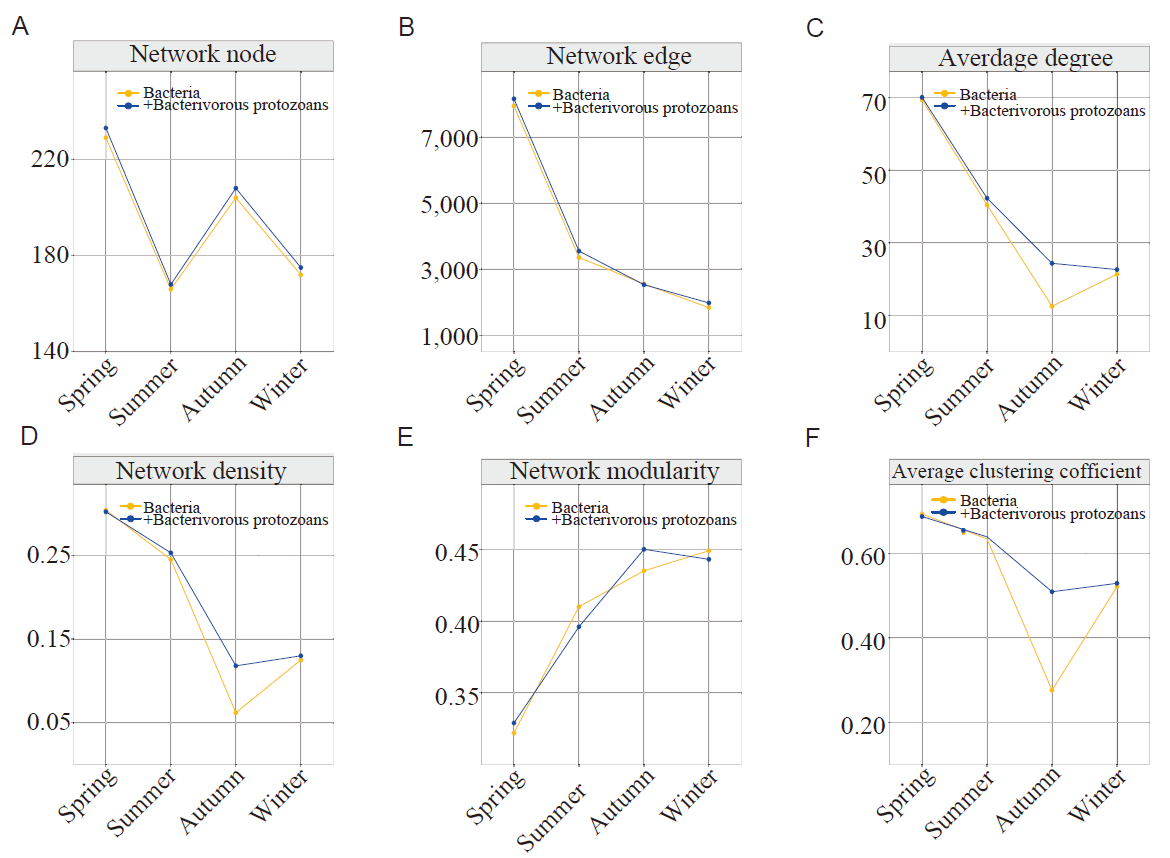


Fig. S8 Topological properties of the co-occurring network in different seasons. (A) network nodes, (B) network edges, (C) average degree, (D) network density, (E) network modularity, (F) average clustering coefficient.


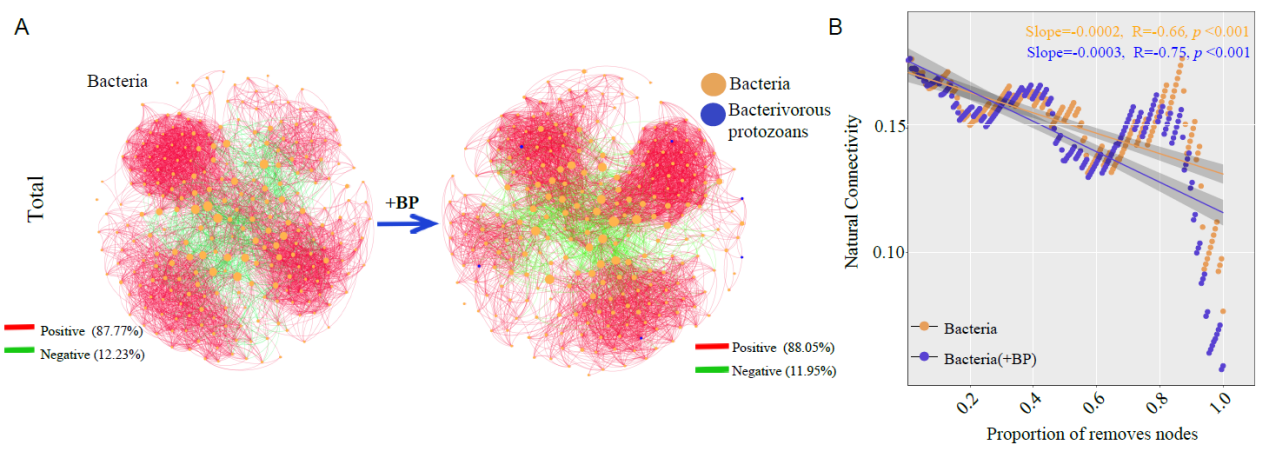


Fig. S9 The co-occurrence network of bacterial community with the addition of bacterivorous protozoans at the whole seasons. (A) Changes in network topology with bacterivorous protozoans addition. (B) Changes in network stability with bacterivorous protozoans addition. R-values and slopes are shown in the diagrams. “+BP” represents the addition of bacterivorous protozoans relationships to the bacterial community.
